# The Late Dr. Davidson’s Library

**Published:** 1888-09

**Authors:** 


WE BEG TO DIRECT the attention of the profession to the valuable library of the late Dr. Davidson, which the executor desires to dispose of at private sale. Very many valuable works are offered on reasonable terms, and we hope the opportunity will be improved by his many friends. We are requested also to state that a large number of unbound medical journals, procured through our exchange list, are available to any of our readers who desire to complete their sets of journals. We earnestly trust that our readers will step forward and assist in the sale of these valuable works.

